# Bias in energy system models with uniform cost of capital assumption

**DOI:** 10.1038/s41467-019-12468-z

**Published:** 2019-10-09

**Authors:** Florian Egli, Bjarne Steffen, Tobias S. Schmidt

**Affiliations:** Energy Politics Group, ETH Zurich, Zurich, Switzerland

**Keywords:** Energy economics, Energy modelling

**Arising from** D. Bogdanov et al. *Nature Communications* 10.1038/s41467-019-08855-1 (2019).

Several studies have recently evaluated the feasibility of 100% renewable energy-based energy systems in different world regions. In a recent article, Bogdanov et al.^1^ contribute to this literature, by using an energy system model that takes into account the unique conditions of 145 global subregions, including factors such as renewable energy (RE) resource conditions, structure and age of existing capacities, demand patterns, etc. Based on their results, they discuss transition pathways and calculate the 2050 levelized cost of electricity generation (LCOE) of 100% RE-based energy systems in those 145 subregions. While the paper provides a new high-resolution analysis of 100% RE systems, we believe that it falls short of adequately considering large differences in the cost of capital (CoC) when comparing the LCOE between countries. As a result, Fig. 2 in Bogdanov et al. shows the lowest LCOEs for solar photovoltaic (PV)-based systems in countries such as the Democratic Republic of Congo (DRC) and Sudan, which seems at odds with the high investment risks and very low installed capacity in both countries^2^. Accounting for CoC differences between countries changes the results dramatically, as we show in Fig. 1. We therefore argue that using uniform CoC can lead to distorted policy recommendations.

**Fig. 1:**
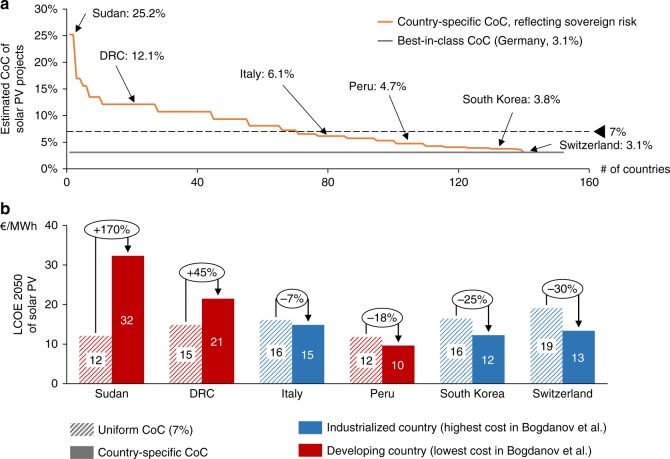
Country-specific solar PV CoC and its effects on 2050 LCOE in six countries with the highest and lowest solar energy system cost as reported in Bogdanov et al. **a** Calculated country-specific CoC for 152 countries based on a risk-free CoC of 3.1% and risk premium according to Moody’s sovereign ratings. **b** Change in 2050 solar PV LCOE due to changes in the CoC. The LCOE calculation shows values for single-axis tracking solar PV systems, which is the most deployed type in Bogdanov et al. Note that we do not perform an energy system calculation but for illustrative reasons focus on the LCOE. Grid and storage infrastructure investments are similarly affected by CoC differences

Wind power, PV, and hydropower are capital intensive, making the CoC a major determinant of these technologies’ LCOE^3–7^. While Bogdanov and colleagues mention CoC as a “major factor of uncertainty”, they assume a uniform CoC of 7% throughout the entire analysis. In reality, however, the CoC strongly varies across countries^8^. While the time value of money might be uniform, the risk premium for long-term investments varies due to differences in macroeconomic stability, political uncertainties, and the maturity of financial markets in different countries^3,9,10^.

Figure 1a shows that risk spreads across 152 countries vary from 0 to 22.1% according to common metrics. Figure 1b illustrates the effect of the country risk spread for the three most expensive (Italy, South Korea, Switzerland) and the three least expensive (DRC, Peru and Sudan) solar PV-based energy systems in 2050 as reported in Bogdanov et al. It compares the 2050 LCOE of solar PV assuming a 7% CoC versus a country-specific CoC. Apart from the CoC, our calculation uses the input parameters from Bogdanov et al. To arrive at the country-specific CoC, we use the 10-year average (2008‒2017) CoC for solar PV in Germany (3.1%)^11^ to establish a lower bound. To this lower bound, we add a country premium corresponding to Moody’s country rating for each country other than Germany (zero premium in the case of Switzerland)^12^.

The results show that solar PV LCOEs are between 7 and 30% lower for the set of industrialised countries and up to 170% higher for the set of developing countries when assuming a country-specific CoC. These numbers are similar and slightly more pronounced when estimating 2015 instead of 2050 LCOEs (−30 to +180%). Importantly, the LCOE in the three industrialised countries are substantially lower than those in DRC or Sudan, when using country-specific CoC, turning the results reported in Fig. 2 of Bogdanov and colleagues upside down. Of the three analysed developing countries, Peru is the only case for which assuming country-specific financing costs would in fact result in lower costs than those reported by Bogdanov and colleagues (−18%). Based on these results, we argue that one can expect the following pattern: 2050 LCOEs of renewables in most developing countries would likely be substantially higher and in most industrialised countries substantially lower than those projected by Bogdanov and colleagues.

The implications are stark. Using uniform CoC may underestimate the cost of RE in developing countries and overestimate it in industrialised countries. Consequently, such analysis conceals the important role of de-risking policies in enabling RE deployment in developing countries^3^. At the same time, the bias may undermine policymakers’ efforts to push forward the RE expansion in industrialised countries, e.g., by pointing to seemingly more cost-efficient options in developing countries. Importantly, our critique of (quasi-)uniform CoC in country-level cost comparisons is not confined to Bogdanov and colleagues but applies to other models, such as the LCOE models of the International Energy Agency (IEA) or the International Renewable Energy Agency (IRENA) too. The IEA uses a CoC of 7% for OECD countries and 8% for the rest^13^. The IRENA uses a CoC of 7.5% for OECD countries and China and 10% for the rest^5^. For modelling and interpreting global outcomes, the use of uniform CoC is not necessarily problematic. However, our above calculations demonstrate that if the models are used to compare country-specific RE LCOEs as an output, they should clearly use country-specific CoC.

Empirically, the CoC also varies between technologies, though technology spreads are small compared with country spreads^8^, especially for mature technologies (which seems a fair assumption for the 2050 scenario). It should also be noted that CoC can change over time—not just in terms of the general interest rate level, but also the risk spreads between countries. There are indeed examples of low- or middle-income countries that were able to reduce their risk spreads compared with industrialised countries to almost zero over a few decades. For example, from 1998 to 2019, South Korea improved its credit rating from Ba1 to Aa2, which corresponds to a 2.3%-point risk-spread reduction^12,14^. However, South Korea is one of the very few countries that have escaped the low- or middle-income traps during the last few decades^15^. Such strong improvements require overcoming many institutional and socioeconomic challenges. A case in point is the persisting large difference in solar PV CoC between Eastern and Western countries of the European Union^8^.

Therefore, we believe that in projection studies such as Bogdanov et al., where no better knowledge is available, the prudent approach is to assume that current economic differences persist, reflected in the respective CoC differences. To study the sensitivity of the model towards this assumption, we also calculate the LCOEs assuming a halved spread between Germany and developing countries. The underestimation of LCOEs in high-risk countries is lower, but differences compared with the uniform CoC remain (+64% for Sudan, +5% for DRC). However, the past has shown that risk spreads can also increase and it is far from certain that developing countries will consistently be able to close the gap to industrialised countries. Uniform CoC (zero risk spreads) assumes global convergence to similar macroeconomic stability, political uncertainty, etc. and should be labelled accordingly.

To conclude, we argue that (renewable) energy system models that compare countries—and particularly countries across different income and investment risk classes—should always employ country-specific CoC. Using uniform CoC may result in distorted results and policy implications.

## Data Availability

The data and the underlying model are available from the authors upon reasonable request.

## References

[1] Bogdanov D (2019). Radical transformation pathway towards sustainable electricity via evolutionary steps. Nat. Commun..

[2] IRENA. *Renewable Capacity Statistics 2018* (2018).

[3] Waissbein, O., Glemarec, Y., Bayraktar, H. & Schmidt, T. *Derisking Renewable Energy Investment* (UNDP, 2013).

[4] Schmidt TS (2014). Low-carbon investment risks and de-risking. Nat. Clim. Change.

[5] IRENA. *Renewable Power Generation Costs in 2017* (2018).

[6] Ondraczek J, Komendantova N, Patt A (2015). WACC the dog: the effect of financing costs on the levelized cost of solar PV power. Renew Energ..

[7] Hirth L, Steckel JC (2016). The role of capital costs in decarbonizing the electricity sector. Environ. Res. Lett..

[8] eclareon & Fraunhofer I. S. I. Re-frame database. http://re-frame.eu/ (2019).

[9] Hail L, Leuz C (2006). International differences in the cost of equity capital: do legal institutions and securities regulation matter?. J. Account. Res..

[10] Acemoglu D, Zilibotti F (2002). Was prometheus unbound by chance? Risk, diversification, and growth. J. Polit. Econ..

[11] Egli F, Steffen B, Schmidt TS (2018). A dynamic analysis of financing conditions for renewable energy technologies. Nat. Energy.

[12] Damodaran, A. Country default spreads and risk premiums. http://pages.stern.nyu.edu/~adamodar/New_Home_Page/datafile/ctryprem.html (2019).

[13] OECD/IEA. *World Energy Outlook 2016* (2016).

[14] Duggar, E. *Sovereign Default and Recovery Rates, 1983–2007* (2008).

[15] Lee K, Malerba F (2017). Catch-up cycles and changes in industrial leadership: windows of opportunity and responses of firms and countries in the evolution of sectoral systems. Res. Policy.

